# COST-EFFECTIVENESS OF THE USE OF ACERTO PROTOCOL IN MAJOR DIGESTIVE SURGERY

**DOI:** 10.1590/0102-672020210002e1660

**Published:** 2022-06-24

**Authors:** José Eduardo de AGUILAR-NASCIMENTO, Alberto BICUDO-SALOMÃO, Mara Regina Rosa RIBEIRO, Diana Borges DOCK-NASCIMENTO, Cervantes CAPOROSSI

**Affiliations:** 1 Departamento de Clinica Cirúrgica do Hospital Universitário Júlio Muller da Universidade Federal de Mato Grosso - MT, Brazil;; 2 Pós-Graduação em Ciências da Saúde da Faculdade de Medicina da Universidade Federal de Mato Grosso - MT, Brazil;; 3 Professora Associada da Faculdade de Enfermagem da Universidade Federal de Mato Grosso - MT, Brazil;; 4 Professora Associada da Faculdade de Nutrição da Universidade Federal de Mato Grosso - MT, Brazil

**Keywords:** Hospital costs, Perioperative Care, Multimodal Treatment, Postoperative Complications, Length of Stay, Custos Hospitalares, Assistência Perioperatória, Tratamento multimodal, Complicações Pós-Operatórias, Tempo de Internação

## Abstract

Hospital costs in surgery constitute a burden for the health system in all over the world. Multimodal protocols such as the ACERTO project enhance postoperative recovery.

**OBJECTIVE:**

The aim of this study was to analyze the hospital costs in patients undergoing major digestive surgical procedures with or without the perioperative care strategies proposed by the ACERTO project.

**METHODS:**

Retrospective data from elective patients undergoing major digestive surgical procedures in a university hospital between January 2002 and December 2011 were collected. The investigation involved two phases: between January 2002 and December 2005, covering cases admitted before the implementation of the ACERTO protocol (pre-ACERTO period), and cases operated between January 2006 and December 2011, after implementation (ACERTO period). The primary outcome was the comparison of hospital costs between the two periods. As secondary end point, we compared length of stay (LOS), postoperative complications, surgical-site infection (SSI) rate, and mortality.

**RESULTS:**

We analyzed 381 patients (239 of the pre-ACERTO period and 142 of the ACERTO period) who underwent major procedures on the gastrointestinal tract. Patients operated after within the ACERTO protocol postoperative LOS had a median of 3 days shorter (p=0.001) when compared with pre-ACERTO period [median (IQR): 10 (12) days vs. 13 (12) days]. Mortality was similar between the two periods. Postoperative complications risk, however, was 29% greater (RR: 1.29; 95%CI 1.11-1.50) in the pre-ACERTO period (p=0.002). SSI risk was also greater in pre-ACERTO period (RR: 1.33; 95%CI 1.14-1.50). Costs (mean and SE) per patients were R$24,562.84 (1,349.33) before the implementation and R$19,912.81 (1,459.89) after the ACERTO protocol (p=0.02).

**CONCLUSION:**

The implementation of the ACERTO project in this University Hospital reduced the hospital costs in major digestive procedures. Moreover, the implementation of this modern perioperative care strategy also reduced postoperative complications, SSI risks, and LOS.

## RESUMO - RACIONAL

Custos hospitalares em cirurgia constituem um peso para o sistema de saúde. Protocolos multimodais como o projeto ACERTO aceleram a recuperação pós-operatória.

**OBJETIVO:**

O objetivo deste estudo foi o de analisar custos hospitalares em pacientes submetidos a procedimentos cirúrgicos de grande porte no aparelho digestivo com ou sem as estratégias de cuidados perioperatórios proposta pelo projeto ACERTO.

**MÉTODOS:**

Foram coletados dados retrospectivos de pacientes eletivos submetidos a procedimentos cirúrgicos de grande porte no aparelho digestivo em um Hospital Universitário entre Janeiro de 2002 e Dezembro de 2011. O estudo envolveu duas fases: Entre Janeiro de 2002 a Dezembro 2005 envolvendo casos internados antes da implementação do protocolo ACERTO (período pré-ACERTO) e casos operados entre Janeiro de 2006 a Dezembro de 2011, após a implementação (período ACERTO). O desfecho primário foi a comparação de custos hospitalares entre os dois períodos. Como desfechos secundários, comparou-se o tempo de internação (LOS), complicações pós-operatórias, taxa de infecção de sitio cirúrgico (ISS) e a mortalidade.

**RESULTADOS:**

Foram analisados 381 pacientes (239 do período pré-ACERTO e 142 do período ACERTO) submetidos a procedimento cirúrgicos de grande porte no trato gastrointestinal. Pacientes operados dentro do protocolo ACERTO apresentaram mediana (IQR) mediana de tempo de internação três dias menor (p=0.001) quando comparados ao período pré-ACERTO (mediana (IQR): 10 (12) vs. 13 (12) dias). A mortalidade foi similar entre os dois períodos. Entretanto, o risco de complicações pós-operatórias foi 29% maior (RR: 1.29; IC95%: 1.11 - 1.50) no período pré-ACERTO (p=0.002). O risco de SSI também foi maior no período pré-ACERTO (RR: 1.33; 95%CI: 1.14-1.50). Custos (media e SE) per paciente foram de R$ 24562,84 (1349,33) antes da implementação e R$ 19912,81 (1459,89) após o protocolo ACERTO (p=0.02).

**CONCLUSÕES:**

A implementação do projeto ACERTO neste hospital universitário reduziu custos hospitalares em cirurgias digestivas de grande porte. Além disso, a prescrição de estratégias modernas de cuidados perioperatórios também reduziu riscos de complicações pós-operatórias e de SSI e o tempo de internação.

## INTRODUCTION

Multimodal protocols became known around the world after the introduction of the so-called “fast-track surgery” Kehlet and Wilmore in 1980s^13^. The authors defined fast-track surgery as a multimodal strategy to care using a combination of epidural or regional anesthesia, minimally invasive techniques, optimal pain control, aggressive postoperative rehabilitation, and postoperative early enteral or oral nutrition^21^.

The central idea was to reduce the stress response and to abbreviate the recovery after surgery. For this, the ERAS group included more protocols such as the shortening of preoperative fast to the fast-track strategy and published various guidelines to approach with different surgical procedures^11^ ^,^ ^14^.

In Brazil, the ACERTO (Aceleração da Recuperação Total Pós-Operatória - Postoperative Enhanced Total Recovery) multimodal protocol was launched in 2005 and was reported first in 2006^1^. A guideline for the implementation of the ACERTO project was published in 2017^7^ after various articles assured the reduction of important end points such as postoperative length of stay (LOS), postoperative complications, and mortality when compared with traditional care^1^ ^,^ ^4^ ^,^ ^6^.

Hospital costs in surgery represent a burden for the health system all over the world^2^ ^,^ ^11^ ^,^ ^19^ ^,^ ^22^. In this context, the use of ERAS strategies of perioperative care has consistently shown that multimodal protocols can reduce costs and improve cost-effectiveness^12^ ^,^ ^18^ ^,^ ^20^. The reduction in hospital costs can be seen not only in major operations but also in hernioplasties in our hospital through the modification from traditional care to the ACERTO protocol^16^. However, until now, we did not have an analysis of costs in major procedures using the ACERTO protocol.

Furthermore, we could not find this analysis in our currency in other studies. We then hypothesized that, as we have initially found in hernioplasties, the cost-benefit should be greatly reduced in major operation with the new implemented multimodal protocol of perioperative care in our university hospital.

Thus, the aim of this study was to analyze the hospital costs in patients undergoing major digestive surgical procedure with or without the perioperative care strategies proposed by the ACERTO project.

## METHODS

This study was submitted for evaluation and approved by the Research Ethics Committee (CEP) of the HUJM (CAAE: 22803019.4.0000.5541) in 2019. Retrospective data were collected from electronic and paper files of elective patients undergoing surgical procedures at the General Surgery Service (Department of Surgery) of the Julio Muller Hospital of the Federal University of Mato Grosso - MT, Brazil, between January 2002 and December 2011. We included in the study only patients who underwent major elective gastrointestinal procedures. Patients transferred for other hospital or having missing data involved in cost analysis were excluded.

The investigation involved two phases: between January 2002 and December 2005, covering cases admitted before the implementation of the ACERTO protocol (pre-ACERTO period), and the other, with cases operated between January 2006 and December 2011, after its implantation (ACERTO period). Table 1 shows the protocols established by ACERTO protocol and the conventional procedures that had been applied before its implementation in the infirmary of the hospital.

**Table 1 -:** Protocols before and after implementing the postoperative ACERTO project.

Conventional protocol (pre-ACERTO period)	ACERTO protocol
No preoperative counseling	Preoperative education
Minimum preoperative fasting of 8 h (from the night before surgery).	Prolonged preoperative fasting not allowed. Indication of carbohydrate-rich liquid diet until 2 h before the operation. Exception: important gastroesophageal reflux, gastroparesis, intestinal obstruction, and clinical or endoscopic evidence of slow gastric empty
Preoperative nutrition therapy as recommended by the dietitian. Postoperative nutritional therapy at the discretion of the surgical staff	Preoperative nutritional therapy for a minimum of 5 days with oral protein supplements or enteral nutrition in all major operations. Nutritional therapy maintained postoperatively
Initiation of postoperative diet after elimination of flatus or bowel movement (patient without “ileum”).	Early postoperative refeeding (the target was to initiate in the same day of operation or the first postoperative day. In operations with esophageal anastomosis, the re-introduction of diet was done by using a feeding catheter (jejunostomy or nasoenteral tube)
Postoperative venous hydration volume of 40 ml/kg. The type of crystalloid fluid was at the discretion of the surgeon	Oral/enteral hydration was the first option. The target of postoperative venous hydration volume was 30 ml/kg/day until the first postoperative day. If oral/enteral nutrition was initiated and tolerated IV hydration was terminated
Systematic mechanical preparation of the colon for colorectal operations with mannitol or phospho-soda	No mechanical bowel preparation except for rectal procedures
Use of drains, nasogastric tube, urinary catheters, and antibiotics according to the preference of the surgeon	Restrict use of abdominal drains. No routine use of nasogastric tube for drainage. Antibiotic prophylaxis for 24/48 h
Early postoperative mobilization at nurse or other staff discretion	Ultra-early mobilization protocol making the patient walk or sit on the same day of operation for at least 2 h and for 6 h in the following days (if possible)

All patients underwent nutritional assessment by the subjective global assessment (SGA), as previously described^1^. In summary, patients bearing score A were considered eutrophic and if scoring B or C they were considered malnourished.

The main end point was the daily total cost of hospitalization, comparing the two periods studied according to the method described below. The hypothesis, formulated prior to data collection, was that patients undergoing the ACERTO perioperative care would have lower daily total costs due to reduced postoperative complications, surgical-site infection (SSI) rate, and a shorter LOS. Accordingly, as a secondary end point, we compared LOS, postoperative complications, SSI rate, and mortality in both periods. Postoperative complications and SSI were defined according to the criteria proposed by Mangram et al.^15^ *.*

### Cost analysis

The primary outcome of the study was the difference in hospital costs between the two periods. We used the costs accrual method according to NBCT 16.11 - Public Sector Cost Information System^5^ ^,^ ^16^. This method allows an indirect calculation of daily cost of the patients as follows. To obtain the average cost of hospitalization per patient per day, we divided the total costs of hospitalization in the infirmary of Surgery Clinics by the patient/day annual average. As for the calculation of the average cost per number of hospitalizations, we divided the total costs of admissions to the Surgical Clinics by the number of hospitalizations performed in each period. Finally, the value of the average cost of hospitalization per night consisted of dividing the total costs of admission to the Surgical Clinic by the number of daily rates in the period.

For the purposes of calculating the average cost of hospitalization at the surgical clinics of the HUJM, we used the following data: (1) product output report by sector issued by the MV 2000 inventory control system; (2) laboratory and image examination report issued by the MV 2000 exam billing system; (3) authorizations for hospital admission (AIH) movement report - reduced files and rejected AIH issued by the DataSUS/Tabwin system; (4) personnel data sheet for public employees provided by the HUJM human resources unit; (5) personnel data from the servants of the single legal regime (RJU) provided by the HUJM expense settlement and payment unit; (6) work schedule available on the HUJM website; (7) information on the number of equipment in the operating room provided by the head of that unit; (8) data of the clinical engineering contract, as well as footage of the HUJM hospital areas made available by the logistics and infrastructure division; and (9) information on accommodation costs obtained by the hospital accommodation indicators monitoring panel and made available by the hospital accommodation unit. We thus obtained the value of R$1,442.86 for the daily cost of a patient operated on our infirmary.

### Statistical analysis

We planned to do an intention-to-treat analysis, meaning the comparison of the two periods disregarding if, especially in the second period, the patient have received or not received the ACERTO protocol. The normality of the continuous variables was assessed with the Kolmogorov-Smirnov test, and the homogeneity of their variances with the Levene test. To compare daily cost and the length of hospital stay, we used the Student’s t-test accordingly. All other continuous variables were compared using the Mann-Whitney U test. We expressed all continuous data as median and interquartile range (IQR) or as mean and standard error (SE) accordingly. We analyzed categorical variables (i.e., surgical complications, SSI, and deaths) using the chi-square test. We adopted a value of p<0.05 as the statistical significance threshold. As a measure of the association strength, we calculated the relative risk (RR), with 95% confidence interval (95%CI). All calculations were performed using the SPSS statistical package version 20.0.

## RESULTS

During the period of the study, 4,071 elective procedures were carried out in the hospital (1,805 patients, 44.3% in the pre-ACERTO period; and 2,266 patients, 55.7% in the ACERTO period). Of these, 2,014 patients were submitted gastrointestinal procedures. We excluded 1,633 patients due to minor procedures such as cholecystectomies, hernioplasties, and anal procedures (n=1452); emergency or urgency operations (n=106); and missing data (n=75). We then analyzed 381 patients who underwent major digestive procedures. Table 2 shows the demographic and clinical variables of these patients according to the two periods of the study. For this study, esophagectomy, any surgical procedure to megaesophagus, total or partial gastrectomy, gastroenteric anastomosis, biliodigestive anastomosis, gastro- or duodeno-pancreatectomy, partial pancreatectomy, colorectal resection, and colostomy closures were considered the major procedures.

**Table 2 -:** Characteristics of the patients operated on in the pre-ACERTO or ACERTO period of the study.

Variable	Pre-ACERTO	ACERTO	Total	p
Patients (n%)	239 (62.7)	142 (37.3)	381	
Sex (n%)				
Males	126 (52.7)	66 (46.5)	192	0.14
Females	113 (47.3)	76 (53.5)	189	
Age (median IQR)	51 (26)	48 (28)		0.30
Malnutrition (SGA-B or SGA-C) (n%)	133 (55.6)	84 (59.2)	267	0.79
ASA score >2 (n%)	31 (13.0)	22 (15.5)	53	0.47
Laparoscopic access (n%)	7 (2.9)	4 (2.9)	11	0.95
Organ (n%)				
Esophagus	29 (12.1)	21 (14.8)	50	
Stomach	53 (22.2)	35 (24.6)	88	0.11
Biliary tree	52 (21.7)	17 (12.0)	69	
Pancreas	12 (5.0)	4 (2.8)	16	
Large bowel	93 (38.9)	65 (45.8)	158	

IQR: interquartile range; SGA: subjective global assessment (scores B and C = malnutrition); ASA: American Society of Anesthesiologists.

### Length of Stay

Patients operated after the implementation of the ACERTO protocol had a median (IQR) postoperative LOS of 3 days shorter (p=0.001) when compared with those from the pre-ACERTO period [10 (12) days vs. 13 (12) days].

### Mortality, Postoperative complications, and SSI

Mortality was 8.4% (n=32) without differences between the two periods [pre-ACERTO=10% (n=24) vs. ACERTO=5.6% (n=8); p=0.13]. Postoperative complications, however, were 29% greater (RR: 1.29; 95%CI 1.11-1.50) in the pre-ACERTO period (p=0.002). Similarly, SSI risks were greater in pre-ACERTO period than the ACERTO period (RR: 1.33; 95%CI 1.14-1.50). These results are shown in Table 3.

**Table 3 -:** Clinical outcome in the two postoperative periods.

Outcome	Pre-ACERTO period N=239	ACERTO period N=142	p
Mortality (n, %)	24 (10.0)	8 (5.6%)	0.13
Postoperative complications (n, %)	81 (33.9)	27 (19.0)	0.002
SSI (n, %)	50 (20.9)	13 (9.2)	0.003
LOS (median, IQR)	13 (12)	10 (12)	0.001

SSI: surgical-site infection; LOS: length of stay; IQR: interquartile range.

### Hospital costs

**Figure 1 -:**
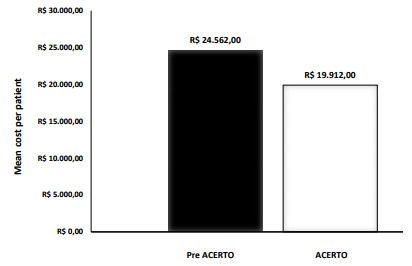
Mean cost in Brazilian reais per patient in the two periods of the study (p=0.02).

The mean (SE) cost of each patient after the implementation of the ACERTO protocol was reduced by almost 20% when compared with before the implementation. The mean reduction in our hospital with 100% patients financed by SUS (Sistema Único de Saúde - Brazilian Public Health System) and submitted to major digestive operations was R$4,650.03. Costs per patients were R$24,562.84 (1,349.33) before the implementation and R$19,912.81 (1,459.89) after the use of the ACERTO protocol (p=0.02) (Figure 1).

## DISCUSSION

Our findings showed that the implementation of a multimodal protocol of perioperative care such as the ACERTO protocol reduces costs in major digestive surgical procedures. These findings probably are associated with the concomitant decrease of postoperative complications and LOS. This is the first study analyzing the reduction of costs with the ACERTO project in major operations using Brazilian currency. Various studies which compared costs with or without ERAS multimodal protocol showed the same with international currencies such as Euros, British Pounds, or Swiss Francs.^12^ ^,^ ^18^ ^,^ ^20^ The overall results seem to agree that changing traditional to modern perioperative care may decrease costs and postoperative morbidity.

We used a strategy of cost analysis using an indirect method that assessed daily total hospital charges as previously described^16^ ^,^ ^19^. The administrative core of the hospital was involved and produces data from the various units of costs, which ail the base of calculations. Our first study with this method was published years ago showing that the type of perioperative care may modify costs in hernioplasties. In the present study, we endorse our first findings showing this time that the reduction in Brazilian Reais is also finding in major operations.

The reduction of LOS and postoperative complications, especially SSI, had already been reported with the ACERTO perioperative care in elective procedures^1^ ^,^ ^3^ ^,^ ^8^. By changing the perioperative nutritional approach by means of a protocol rather than the staff criteria probably had an important role in these results. Moreover, the decrease of preoperative fasting to 2 h, only used after the implementation of the new protocol, may have also contributed to the better results^9^. Decreasing the fast time before and after the surgical procedure reduced the organic response to trauma and decrease not only the LOS but also postoperative complications in various studies^4^ ^,^ ^9^. Nutritional attention is mandatory and should be implemented in these patients according to many guidelines based on evidence^7^ ^,^ ^10^ ^,^ ^14^.

Pimento et al. have recently reported that healthcare costs can be reduced by the implementation of nutrition intervention for patients with gastrointestinal cancers^17^. We agree with that, since the implementation of the ACERTO project changed the nutritional approach in our patients from a staff-oriented perspective to a protocol-oriented protocol, which was absorbed by the multidisciplinary staff of the hospital including not only surgeons but also nurses, dietitians, and physiotherapists.

However, our findings have limitations. First, hospital daily charges were assumed to be an accurate surrogate for hospital costs. More accurate methods may be available, but we were unable to access. Second, as a retrospective study, there is a time-dependent bias, which can lead to overestimating or underestimate the financial impact^19^. Although all patients were from the same public health system and were operated on a single center, and with the same surgeons, the data may also have bias due to the different number of patients undergoing major operations between the two periods.

The new public hospitals in various cities of Mato Grosso State along with new surgery residency programs may have contribute to a decrease in the number of major operations during the second period of the study. We believe that patients bearing surgical oncological digestive diseases once referred to our University hospital were operated on in new hospitals during the last period. However, we compared only major procedures in the two periods. Due to this, we considered our findings as appropriate to be compared though the total number of procedures in the two periods were uneven. A decrease in costs using ERAS protocol by comparing two periods as we used in this study has been reported as well, which is consistent with our findings^12^ ^,^ ^20^.

## CONCLUSION

The implementation of the ACERTO project in the University hospital, including only patients bearing the Brazilian public health system insurance, reduced the hospital costs in major digestive procedures. Moreover, the implementation of this modern perioperative care strategy also reduced postoperative complications, SSI, and LOS.
